# scCAPReSE: detection of large-scale genomic rearrangements from single-cell Hi-C based on few-shot learning

**DOI:** 10.1186/s44342-026-00069-4

**Published:** 2026-03-16

**Authors:** Kyukwang Kim, Chul-Hwan Lee, Inkyung Jung

**Affiliations:** 1 Department of Biological Sciences, Korea Advanced Institute of Science and Technology, Daejeon, Republic of Korea; 2 Department of Biomedical Sciences and Pharmacology, Seoul National University College of Medicine, Seoul, Republic of Korea; 3 Cancer Research Institute, Ischemic/Hypoxic Disease Institute, Medical Research Center, Seoul National University, Seoul, Republic of Korea; 4 Wide River Institute of Immunology, Seoul National University, Hongcheon, Republic of Korea

**Keywords:** Structural variation, 3D genome, Cancer, Single-cell Hi-C, Few-shot learning, Foundation model

## Abstract

Large-scale genomic rearrangements are prevalent in cancer genomes and can profoundly rewire three-dimensional (3D) genome architecture, leading to aberrant oncogene activation through enhancer hijacking. The rewired 3D organization generates unique chromatin contact signatures, which can be detected using deep learning-based approaches. However, extending such analyses to single-cell resolution, which is critical to delineate clonal heterogeneity in cancer, remains a major challenge, due to the limited number of training sets as single-cell Hi-C techniques are not standardized and only limited datasets are available across different methods. Here, we introduce scCAPReSE, a few-shot learning-based framework that adopts representations from a pre-trained image foundation model, CLIP, to enable robust classification of structural variation (SV) patterns in single-cell Hi-C data. By extracting and fine-tuning base weights from the foundation model, scCAPReSE enables effective training of deep learning classifiers using only a few hundred large-scale SV examples derived from a single cancer cell line while adapting classification tasks to heterogeneous single-cell Hi-C libraries. scCAPReSE achieved over 90% classification accuracy when evaluated on sci-Hi-C datasets. When further applied to scNanoHi-C data from the K562 chronic myeloid leukemia cell line, scCAPReSE correctly identified the Philadelphia chromosome translocation but also revealed substantial cell-to-cell variability in the contribution of SV-mediated chromatin interactions, highlighting previously inaccessible heterogeneity in cancer 3D genome organization. In summary, scCAPReSE provides a broadly applicable and data-efficient framework for detecting SV-driven 3D genome reorganization at single-cell resolution, enabling quantitative dissection of cancer-specific chromatin architecture and clonal heterogeneity. The developed method is freely available at https://github.com/kaistcbfg/CAPReSE.

## Introduction

Cancer genomes frequently harbor large-scale structural variations (SVs), such as deletions, inversions, duplications, and chromosomal translocations [1], which can profoundly disrupt genome organization and gene regulation [2, 3]. Beyond altering linear DNA sequence, these SVs reshape the three-dimensional (3D) genome architecture, thereby rewiring regulatory interactions and contributing to oncogenic processes, including enhancer hijacking and aberrant gene activation [4–7]. Disruption of 3D genome organization by large-scale genomic rearrangements or structural variations (SVs) enables Hi-C as a powerful complementary method for SV detection [8]. Compared to whole-genome sequencing (WGS), Hi-C not only detects SVs but also visualizes the SV-mediated aberrant 3D genome, providing a starting point for interpreting the functions of SVs occurring in noncoding regions [9].

To utilize these features, Hi-C-based SV detection tools have been developed. Early approaches primarily relied on statistical frameworks to identify aberrant 3D genome patterns [10, 11], whereas more recent methods leverage deep learning techniques [9, 12]. Although deep learning-based Hi-C SV detection methods have shown excellent performance with low false-positive rates, they typically require large amounts of data for training. Common deep learning benchmark datasets such as MNIST and CIFAR10 provide approximately 60,000 training samples, while IMAGENET contains over 1 million images. In contrast, even a large-scale cancer cohort yields only on the order of 1000 large-scale SV patterns [9], and their validation often requires additional whole-genome sequencing data. Compared to other omics data with relatively large amounts of accumulated data [13, 14], the amount of cancer Hi-C data with matching WGS is still insufficient. Furthermore, organizations on the Hi-C contact map are affected by sequencing depth, making it challenging to construct models that generalize across datasets with varying coverage [15]. The problem of lacking uniform training data is further exacerbated in single-cell Hi-C. Although several single-cell Hi-C protocols have been developed [16–20], each produces limited data and lacks a unified standard, thereby restricting the application of deep learning-based approaches to cancer 3D genome analysis at the single-cell level.

The emergence of transformer architecture [21] and the construction of a foundation model based on self-supervised learning [22] have advanced the performance of deep learning models [23–26]. In line with these developments, recent methods leverage pre-trained weights from foundation models instead of training models entirely from scratch. For example, the pre-trained Contrastive Language-Image Pre-training (CLIP) model [27] uses contrastive learning to align outputs from the vision and text encoders to similar values, which allows CLIP to perform various tasks, such as zero-shot classification, text-based image generation [28], and model evaluation [29].

By exploiting CLIP’s strong visual representations, few-shot learning strategies that effectively learn from limited data can be implemented. Here, we adopt this approach to classify SV patterns from single-cell Hi-C data using a small number of available samples. We developed a few-shot learning algorithm optimized for SV pattern classification by combining the Tip-Adapter method, which fine-tunes CLIP features as the weights of a neural network, with a pre-established SV detection image processing algorithm [30]. We demonstrated classification performance by applying this to a public single-cell Hi-C library and further showed that the SV-mediated 3D genome alterations caused by chromosomal translocation vary across individual cells.

## Materials and methods

### Bulk Hi-C dataset

Bulk cancer Hi-C datasets were prepared to construct the base weight of the Tip-Adapter few-shot learning method. A total of nine cancer cell lines (A549, SK-N-MC, LNCaP, PANC-1, NCI-H460, Caki2, T47D, K562, and MCF7) with WGS-identified SV data used in the previous studies were used as the positive set [12, 31, 32]. The negative set was generated by using two normal cell lines (“WI38_RAF uninduced” and “IMR90 control vector”) and two peripheral blood T-cells (“1” and “3”) Hi-C data. All Hi-C data were downloaded from the 3DIV database (notation indicates 3DIV listed names [33, 34]; original sources are available at the 3DIV homepage). All bulk Hi-C data were processed into 40-kb resolution and normalized by the covNorm R package [35].

The Hi-C signal clusters detected by the image preprocessing algorithms were collected and used as the negative set. Using the property of covNorm [35], which scales overall Hi-C contact map values during normalization, the input Hi-C contact map is capped with the given threshold value (value of 5 used) and converted to a 0–255 scale grayscale image. Then, Otsu’s method is applied to threshold the image, and the majority filter is applied to yield high-strength contours. Filters implemented by the Python “mahotas” package (1.4.13) were used. According to the published Hi-C-based SV detection method, the contours are filtered, but the breakpoint candidates are determined by searching for the corners of the maximum sum sub-matrices. For better classification, low coverage region masks and high-intensity pixel values were further added to the image as an extra channel.

### Single-cell Hi-C dataset

Two libraries (ML1 and ML4) from the single-cell combinatorial indexed Hi-C (sci-Hi-C) and K562 cell line data from the scNanoHi-C were used [17, 20]. The authors provided valid pairs from the sci-Hi-C that were parsed to extract human interactions (HeLa S3 and HAP1 cell lines) into 40-kb resolution from the libraries. The same image preprocessing algorithm applied to the bulk Hi-C data was used on the single-cell Hi-C data to extract candidate Hi-C crops, and the collected crops were manually classified into positive and negative sets. The crops obtained from the ML4 library with the larger number of cells (3182 for ML1 and 8184 for ML4) were used as a training set for the Tip-Adapter, while the crops from the ML1 library were used as a test set. To increase the number of the training set, image augmentation was applied by rotating the Hi-C contact map crop three times by 90°. As a result, we prepared 284 true and 304 false fine-tuning training data. For testing, 33 true data points and 112 false data points were prepared.

In the case of scNanoHi-C, a total of 261 cell-wise cooler formatted files (*.cool) were downloaded. The “merge” function of the cooler command line interface tool was used to generate a 40-kb resolution pseudo-bulk Hi-C contact map.

### CLIP model and Tip-Adapter

The CLIP model was obtained from the official repository of OpenAI (https://github.com/openai/CLIP). For the pre-trained vision transformer (ViT) of the CLIP model, “ViT-B/32” was used, which converts the given image to a 512-length embedding. The Tip-Adapter method was implemented by referring to the published code from the official repository (https://github.com/gaopengcuhk/Tip-Adapter).

### Implementation details

The developed method was developed and tested under the CentOS environment (7.9 minimal). The “mesa-libGL” library was pre-installed using the “yum” installer of CentOS. Using the conda's (2023.09 version) “pip” tool, all Python libraries were installed and managed. Using the Python version of 3.10.13, packages including opencv-python (4.8.1), scikit-learn (1.3.2), Pytorch (2.1.1), and CLIP (1.0.dist) were used. To avoid the version conflict during the installation, the packages were installed in fixed orders. Further details of the environment setup are available at the GitHub repository (https://github.com/kaistcbfg/CAPReSE). Binary files, including the QEMU qcow2 image, are available at the online repository (http://junglab.kaist.ac.kr/Dataset/CAPReSE_scHiC). The train and test phase example codes available on the GitHub repository were tested on the virtual machine with 4 vCPUs and 8 GB RAM without using a GPU. The host machine used Intel(R) Core(TM) i9-7920X CPU at 2.90 GHz.

## Results

### Overview of the single-cell CAPReSE method

**Fig. 1:**
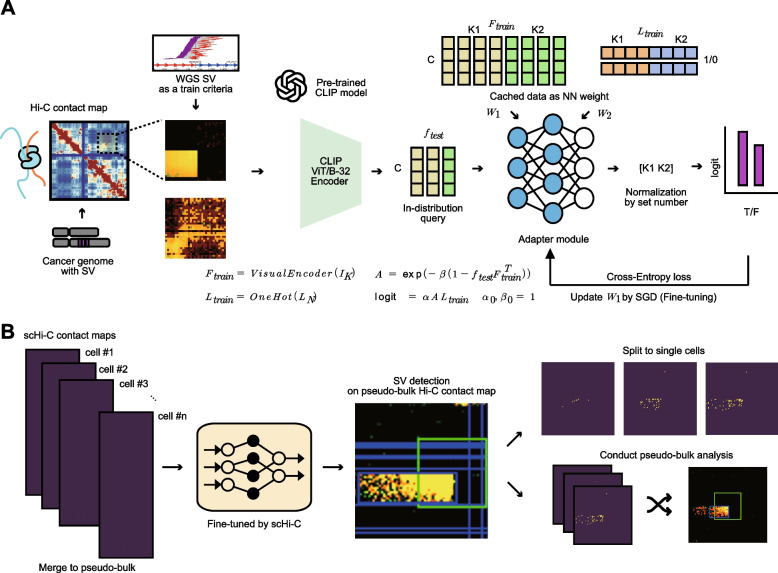
Schematic overview of the few-shot learning-based SV pattern detection in a single-cell Hi-C dataset. **A** Workflow of the few-shot learning-based SV detection method. **B** Strategy for SV pattern detection in single-cell Hi-C dataset

To enable robust detection of SV patterns from pseudo-bulk single-cell Hi-C contact maps under limited training data, we developed a few-shot learning framework (Fig. 1A) named “single-cell chromatin anomaly pattern recognition and size estimation” (scCAPReSE). As the core algorithm, we adopted the Tip-Adapter method, which utilizes the pre-trained CLIP model to adapt general visual representations to domain-specific classification tasks.

SV candidate regions were first identified by scanning contours in Hi-C contact maps using an image-processing-based algorithm (see the “Materials and methods”). SV breakpoints-centered crops for each contour were prepared and processed by the CLIP model to extract image embedding. The original CLIP model conducts zero-shot learning by calculating similarities between the image embedding and text embedding; however, as the CLIP model lacks pre-training for text terms such as “structural variation” or “Hi-C contact map,” proper zero-shot learning cannot be conducted. The Tip-Adapter method was utilized to solve this issue by using a small (two-layer) fully connected neural network (adapter module). After obtaining an example dataset for the classes to be classified, CLIP embeddings of the example dataset (*F*_train_) are used as the weight of the first layer (*W*_1_), while the one-hot labels of the example dataset (*L*_train_) are used as the weight of the second layer (*W*_2_). When the new input data or query (*I*_K_) is given, the logit value is obtained from the adapter module by measuring the CLIP feature similarities between the cached example data and query (*f*_test_). After normalizing the logit with the class number of the original sets (*K1* and *K2* for positives and negatives), the final classification result can be obtained.

Although few-shot learning can be implemented to some extent by measuring CLIP feature similarity with the example dataset, if the query or example images are not used in CLIP pre-training, appropriate CLIP features may not be generated. As the adapter module is a neural network, the stochastic gradient descent (SGD) combined with a cross-entropy (CE) loss can be applied to optimize the weights of the first layer (*W*_1_) to yield better logit results. Note that the weights of the second layer (*W*_2_) are not updated, as *W*_2_ is a simple one-hot vector for the logit calculation.

The original Tip-Adapter uses an equal number of example datasets per class to achieve *K*-shot *N*-class training. As our SV classification task is a binary classification problem and does not require the use of a fixed number of *K* samples, *K1* and *K2* normalization was introduced in this method to allow the use of unbalanced cached data. The text encoder’s output is also utilized in the original process of the Tip-Adapter (Knowledge Incorporation step). As the proper text label was not available, this step was removed during the implementation. Stepwise search for the best hyperparameter (*α* and *β*) was also removed, and an initial value of 1 (no change) was given to *α*_0_ and *β*_0_. Through this process, a few-shot learning pipeline optimized for SV pattern classification was implemented.

### A two-step strategy for detecting SVs in single-cell Hi-C data

SV-driven signatures in single-cell Hi-C contact maps are often indistinguishable at the individual-cell level due to the limited amount of genomic material available per cell, low sequencing depth, limited captured chromatin contacts, and low experimental efficiency. We therefore developed a two-step strategy in which single-cell Hi-C contact maps were first aggregated to generate a pseudo-bulk Hi-C contact map. SV patterns were robustly detected at this pseudo-bulk level and subsequently projected back to individual cells by examining interaction signatures within the identified cancer-specific contact regions (Fig. 1B). This approach enabled reliable detection of SV signatures that were otherwise obscured in sparse single-cell contact maps.

**Fig. 2:**
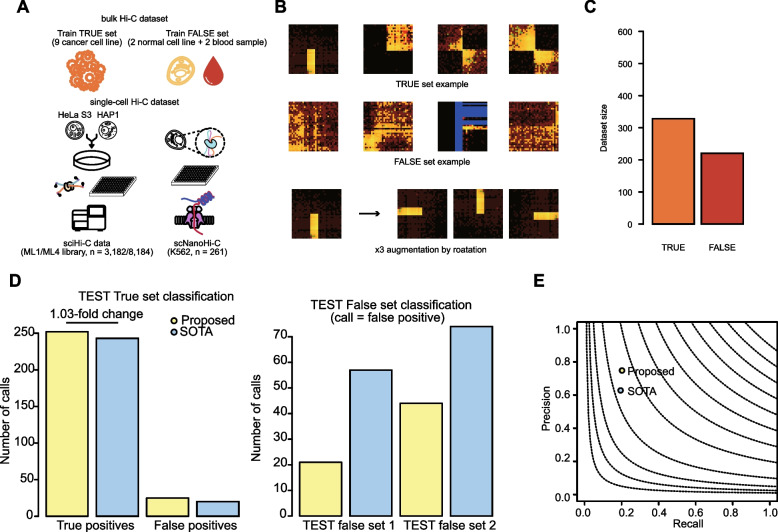
Construction of the train and evaluation dataset. **A** Illustration showing the used bulk and single-cell Hi-C dataset. **B** Examples of positive and negative datasets with image augmentation examples. **C** Bar plot showing the number of collected cached examples for the base-weight construction. **D** and **E** Benchmark results using bulk Hi-C data and WGS-based SV data. Bar plots show the true set (left) and false set (right) classification results (**D**). Overall results are summarized to a precision-recall plot (**E**)

We observed that the effective depth and quality of pseudo-bulk Hi-C contact maps varied depending on experimental protocols and the number of contributing cells. To accommodate this variability, SV patterns derived from bulk Hi-C contact maps, where SV signatures were consistently detectable above a defined sequencing depth, were used to initialize the weights of the adapter module. These weights were then further optimized for single-cell Hi-C SV pattern classification using a few-shot learning framework (Fig. 2A). To expand the diversity of the training and fine-tuning datasets, we applied 90° rotation-based image augmentation (Fig. 2B), yielding a final weight matrix trained on approximately 600 bulk Hi-C SV patterns (Fig. 2C). Together, this strategy enabled robust SV pattern classification across single-cell Hi-C datasets with heterogeneous depth and quality.

### Benchmark of the proposed method

The developed scCAPReSE was designed to be applied to single-cell Hi-C data, but since it uses a pseudo-bulk-based detection strategy, a benchmark was performed using bulk Hi-C data. As state-of-the-art (SOTA) method for the benchmark, EagleC [12], a CNN ensemble-based SV pattern classifier, was used. For comparison based on datasets not used for the model training, we used Hi-C and WGS data generated from tumor and normal tissues of a colorectal cancer patient cohort. A total of 17 tumor tissue data with 17 well-curated WGS-based SV data and 10 normal colon tissue data were used [9]. To supplement the normal Hi-C data, six differentiating stem cell Hi-C data were added [36]. The Hi-C contact map of the normal sample was used as a negative sample to determine whether false-positive classification occurred due to factors such as clutter. Compared to the SOTA method, the scCAPReSE showed 1.03-fold higher accuracy, but the false positives from the test true set also slightly increased (Fig. 2D). However, the proposed method showed a much lower number of calls in normal colon tissue and stem cell data, showing the increase of overall precision and recall values (Fig. 2E).

### Few-shot learning optimization enables robust SV pattern classification

**Fig. 3:**
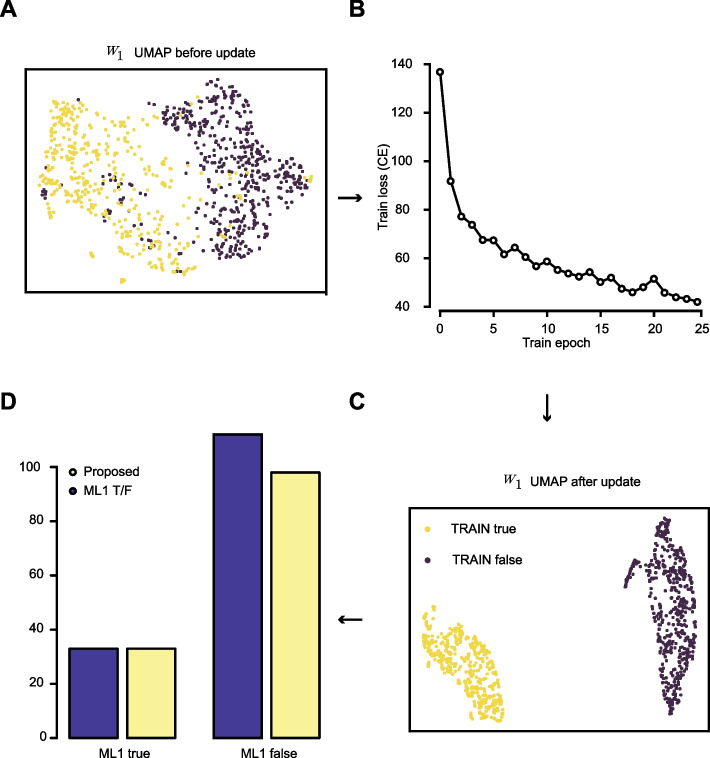
Few-shot learning and evaluation using single-cell Hi-C data. **A** UMAP plot showing cached base weight before fine-tuning. **B** Line plot showing a decrease of the cross-entropy (CE) loss during the fine-tuning process. **C** UMAP plot showing updated base weight after fine-tuning. **D** Bar plot showing the evaluation result after fine-tuning

The pre-established weights of the first adapter layer (*W*_1_) were further optimized to improve SV pattern classification in single-cell pseudo-bulk Hi-C data. Prior to optimization, the weights corresponding to positive and negative SV patterns were closely positioned in the UMAP latent space, with partial overlap between the two classes (Fig. 3A). Such a weight configuration makes it difficult to accurately classify the two classes. Using single-cell Hi-C data, we fine-tuned the adapter weights for 25 epochs, resulting in an approximately 3.5-fold reduction in cross-entropy loss (Fig. 3B). Following optimization with stochastic gradient descent (SGD), the updated weights were segregated into two well-separated and distal clusters in the UMAP space (Fig. 3C), indicating improved class discrimination. Consistent with this separation, classification on the held-out test set achieved near-complete recovery of true SV patterns, with only ~20 false positives (Fig. 3D). Given the limited size of the training dataset, which is largely infeasible with conventional deep learning training approaches, these results demonstrate that combining foundation models with few-shot fine-tuning enables near-optimal SV pattern classification from the single-cell Hi-C data.

**Fig. 4:**
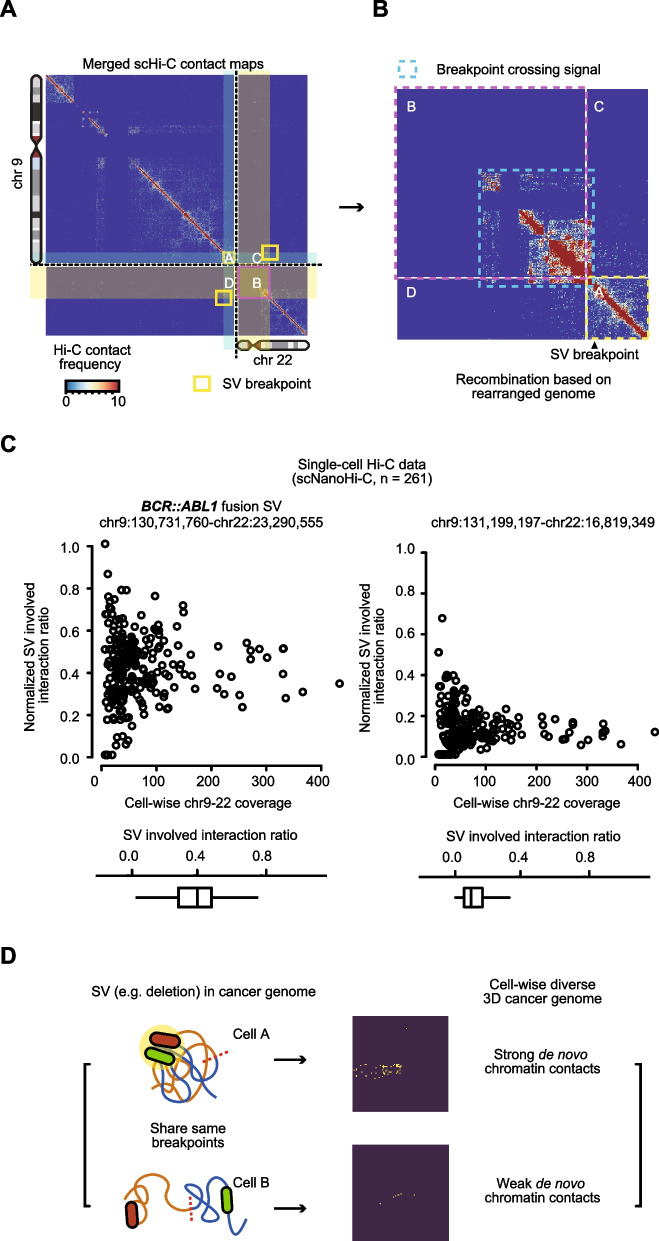
Identification and single-cell level analysis of SV-mediated de novo chromatin contacts. **A** Hi-C contact map showing identified translocation between chromosome 9 and 22 (yellow boxes). **B** Recombined Hi-C contact map showing the breakpoint crossing signal of the identified SV. **C** Scatter plots showing depth-normalized ratio of SV-involved interaction for each cell in scNanoHi-C data. Boxplots at the bottom summarize the distribution. Left, plots of *BCR::ABL1* fusion SV. Right, another WGS-supported translocation between chromosome 9 and 22. **D** Illustrations describing cell-wise diverse 3D cancer genome configuration at the SV-mediated de novo chromatin contacts

We next applied the optimized framework to an independent single-cell Hi-C dataset generated using scNanoHi-C to evaluate its ability to resolve cancer-specific 3D genome configurations at single-cell resolution. To this end, we analyzed scNanoHi-C data from the K562 cell line, which harbors the well-characterized t(9;22) translocation (Philadelphia chromosome) that generates the *BCR::ABL1* fusion gene [37]. The method accurately identified the known translocation from the pseudo-bulk Hi-C contact map (Fig. 4A). Breakpoint-crossing interaction signals, which are indicative of newly formed chromatin contacts between rearranged chromosomes, were clearly visualized (Fig. 4B). Detecting SV patterns on the pseudo-bulk Hi-C contact map enabled downstream analyses of cancer-specific 3D genome features that have previously been restricted to bulk Hi-C data [38].

To resolve these aberrant interactions at the individual cell level, we quantified SV-associated interactions for each cell within the identified rearranged 3D genome. Because sequencing depth varied across individual cells, interaction counts were normalized by the total number of chromosome 9–22 inter-chromosomal interactions per cell. After normalization, cells exhibited a broad distribution of contribution values, with a median of approximately 0.4 (Fig. 4C, left). To examine cell-level variance of SV-involved interactions in different SVs, we also applied the analysis to another translocation between chromosome 9 and chromosome 22 (chr9:131199197–chr22:16819349), which is strongly visible on the Hi-C contact map and supported by WGS. Surprisingly, although both SVs generate interaction patterns on the same Hi-C contact map, the ratio of SV-involved interactions between the two SVs highly differed (Fig. 4C). Even considering the area of the 3D genome altered by SVs (the Philadelphia chromosome-inducing SV was 2.5 times larger), ~4-fold difference in median interaction ratio and ~2-fold dispersion in ratio were observed (Fig. 4C, right). Overall, this variability indicates that despite the presence of a cell line’s signature SV, individual cells exhibit heterogeneous 3D genome configurations (Fig. 4D), even within a relatively homogeneous cancer cell line.

Together, these results demonstrate that learning from a limited number of SV patterns in sparse single-cell Hi-C libraries is feasible using a few-shot learning-based framework, and that this approach enables quantitative assessment of SV-driven 3D genome heterogeneity in cancer.

## Discussion

Biological samples, particularly clinical datasets, often suffer from limited sample sizes, posing a major challenge for training deep learning models. Advanced architectures such as transformer-based models typically require even larger training datasets, as attention mechanisms lack strong inductive bias [39], paradoxically making them less effective for complex tasks with small sample sizes. In this study, we address this limitation by utilizing pre-trained representations from an image foundation model as weights of the adaptor neural network, which serves as a few-shot learning module to classify SV patterns in single-cell Hi-C contact maps. By introducing light-weighted neural network adapters and fine-tuning the foundation model, we demonstrate robust learning performance even with only a few hundred available samples. For typical deep learning models, additional training due to changes in the dataset, such as fine-tuning, requires significant costs, such as GPU resources. Despite the heterogeneity of the bulk Hi-C and single-cell Hi-C models used as base weights, the proposed method successfully trained GitHub examples quickly (~1 min) using desktop-level resources (4 CPUs & 8 GB RAM) without a GPU. Users of scCAPReSE can quickly update base weights when existing learning models do not perform well due to differences in detailed conditions such as the depth of bulk/single-cell Hi-C data or experimental techniques.

Clonal evolution is a defining feature of cancer, in which sequential subclonal selection drives the expansion of genetically and epigenetically distinct tumor cell populations, resulting in profound intra-tumoral heterogeneity [40, 41]. Our previous work demonstrated the inference of clonality of SV-mediated aberrant 3D genome by integrating bulk Hi-C data with WGS; however, analyses at the resolution of individual cells or clones have remained inaccessible due to technical and analytical limitations [9]. The strategy presented here, detecting SV patterns in pseudo-bulk Hi-C contact maps using few-shot learning and subsequently resolving these patterns at the single-cell level, provides a new approach for interrogating cancer clonality beyond the constraints of bulk measurements.

Resolving clonal evolution at single-cell resolution is particularly critical because distinct subclones can harbor SVs with markedly different oncogenic consequences. In particular, SV-mediated enhancer hijacking has emerged as a powerful driver of tumor progression, and our previous work has shown that clonal enhancer hijacking events often exert stronger oncogenic effects than subclonal or passenger rearrangements [9]. Bulk analyses obscure this clonal context, limiting the ability to accurately link regulatory rewiring to tumor fitness, progression, and therapeutic response. These observations highlight the necessity of single-cell-resolved approaches to map SV architectures and their functional impact within heterogeneous tumors.

Single-cell Hi-C offers a uniquely powerful solution to this challenge. Unlike WGS, which detects SVs primarily through breakpoint signals, Hi-C acts as a molecular range finder by capturing long-range physical interactions across the genome [8]. This enables the detection of SV-induced regulatory rewiring events, such as altered enhancer-promoter communication, which may be invisible or ambiguous at the sequence level. Extending Hi-C to single-cell resolution further allows direct interrogation of clonal and subclonal 3D genome configurations, making it possible to disentangle SV-driven regulatory programs across evolving tumor cell populations.

Looking forward, the integration of few-shot learning-based SV detection with emerging multi-omics single-cell Hi-C technologies [42] is expected to enable systematic dissection of how SV-driven 3D chromatin reorganization shapes clonal fitness, transcriptional programs, and therapeutic vulnerability in cancer. Although current limitations in multi-omics single-cell Hi-C data from primary tumors restrict immediate validation, frameworks such as scCAPReSE establish a critical methodological foundation for future studies, enabling regulatory consequences of clonal SVs to be resolved at single-cell resolution and directly linked to oncogenic phenotypes.

## Data Availability

The developed method is freely available at https://github.com/kaistcbfg/CAPReSE. Model weights are available at the online repository (http://junglab.kaist.ac.kr/Dataset/CAPReSE_scHiC).
